# Emerging Roles of DYRK Kinases in Embryogenesis and Hedgehog Pathway Control

**DOI:** 10.3390/jdb5040013

**Published:** 2017-11-21

**Authors:** Rajeev Singh, Matthias Lauth

**Affiliations:** Philipps University Marburg, Institute of Molecular Biology and Tumor Research (IMT), Center for Tumor and Immune Biology (ZTI), Hans-Meerwein-Str. 3, 35043 Marburg, Germany; singhr@staff.uni-marburg.de

**Keywords:** hedgehog, GLI1, dual-specificity tyrosine-regulated kinase, DYRK, MIRK, Down syndrome

## Abstract

Hedgehog (Hh)/GLI signaling is an important instructive cue in various processes during embryonic development, such as tissue patterning, stem cell maintenance, and cell differentiation. It also plays crucial roles in the development of many pediatric and adult malignancies. Understanding the molecular mechanisms of pathway regulation is therefore of high interest. Dual-specificity tyrosine phosphorylation-regulated kinases (DYRKs) comprise a group of protein kinases which are emerging modulators of signal transduction, cell proliferation, survival, and cell differentiation. Work from the last years has identified a close regulatory connection between DYRKs and the Hh signaling system. In this manuscript, we outline the mechanistic influence of DYRK kinases on Hh signaling with a focus on the mammalian situation. We furthermore aim to bring together what is known about the functional consequences of a DYRK-Hh cross-talk and how this might affect cellular processes in development, physiology, and pathology.

## 1. Introduction

### 1.1. The Hedgehog Signaling Pathway

The *Hedgehog (Hh)* gene was first identified in genetic screens for mutations that disrupt the larval body plan in *Drosophila melanogaster* [1]. The name Hedgehog originates from the short and ‘spiked’ phenotype of the cuticle of the Hh mutant *Drosophila* larvae, which resembles the spikes of a hedgehog [1,2]. The members of the Hh family of proteins have since been recognized as key mediators of many fundamental processes in embryonic development, playing a crucial role in controlling cell fate, patterning, proliferation, survival, and differentiation. Furthermore, Hh signaling also regulates the maintenance of tissue stem cells and affects oncogenic transformation and the development of tumors [3,4,5]. Vertebrates possess three Hedgehog homologues: Desert (*DHH*), Indian (*IHH*), and Sonic (*SHH*). All three genes have evolutionary conserved roles in body plan organization and development [2,6,7,8]. The polarizing activity of the organizing centers located in the limb bud, the notochord, or the floor plate of the neural tube is regulated by SHH [9,10]. IHH regulates the coordination of multiple cellular events during endochondral bone development including osteoblast differentiation [11,12], while DHH is required for the development of germ cells in testes and peripheral nerve sheath formation [13]. 

The Hh signaling cascade has been discussed in depth by other excellent reviews of this special issue on embryogenesis (e.g., [14,15,16,17]). Briefly, the canonical Hh signaling cascade is initiated in the target cell by the Hh ligand binding to the Patched1 receptor (PTCH1) [18,19], a 12-span transmembrane protein located in the ciliary membrane relieving the repression of Smoothened (SMO) [20,21], a 7-span transmembrane protein, which is a member of the G protein-coupled receptor (GPCR) superfamily. This de-repression results in the activation of the Hh transcriptional effectors, the zinc finger proteins of the GLI (Cubitus interruptus (Ci) in *Drosophila melanogaster*) family [22]. 

Several studies have reported the modulation of Hh signaling through protein kinases, amongst others PKA, PKC, GRK2, MEK, ERK, AKT, S6K, and GSK3β all of which have been documented to play a role in Hh signal transduction [23,24,25,26,27,28]. Moreover, recent studies have outlined the importance of dual-specificity tyrosine phosphorylation-regulated kinases (DYRKs) in the positive and negative regulation of Hh pathway activity [29,30,31,32,33,34,35,36]. This review centers on the DYRK family of kinases and their role in regulating the developmentally important Hh signaling pathway.

### 1.2. Protein Kinases: An Introduction

Protein kinases are central for the regulation of major cellular processes. Kinases play particularly prominent roles in signal transduction as they direct the cellular activities by the addition or removal of a phosphate group. As abnormal levels of protein phosphorylation are associated with the development of several diseases [37], it is crucial to delve deeper into the understanding of the varying mechanisms that control these phosphorylation events [38]. Eukaryotic protein kinases (ePKs) are divided into nine large groups (plus one atypical group which does not show similarity to ePKs), which are further divided into families and subfamilies [39,40]. These groups are: (1) Tyrosine kinases (TK); (2) Tyrosine kinase-like (TKL); (3) cAMP-dependent protein kinase, cGMP-dependent protein kinase and protein kinase C (AGC); (4) Calcium/calmodulin-dependent kinases (CAMK); (5) Casein kinase 1 (CK1); (6) Cyclin-dependent kinases (CDK), Mitogen-activated protein kinases (MAPK), Glycogen synthase kinase (GSK3) and CDC-like kinase (CLK) group of protein kinases (CMGC); (7) Homologs of the yeast STE7, STE11 and STE20 genes (STE); (8) Receptor Guanylate Cyclases (RGC); and (9) Others (kinases that do not fit within any of the other main kinase groups) [40,41,42].

## 2. The CMGC Group of Kinases

Due to sequence homologies in their kinase domains, CDKs, MAPKs, GSK3s, CLKs, and related kinases (CMGCs) form one big group of eukaryotic protein kinases [40]. The CMGC group consists of 62 members in total, which are subdivided further into nine families (CDK-, CDKL-, GSK-, CLK-, MAPK-, HIPK-, DYRK-, RCK-, and SRPK-families) [43]. This group is highly conserved during evolution, arguing that its members fulfill important functions from nematodes to humans. Given their involvement in cell proliferation, MAPKs and CDKs are the most studied kinases within the CMGC group and are the subject of intense research efforts in oncological research. Less studied candidates include the dual-specificity tyrosine regulated kinases (DYRKs) and the serine-arginine protein kinases (SRPK). In general, the kinases in the CMGC group have a broad spectrum of functional roles ranging from signal transduction to cell cycle regulation, RNA related processing, and intracellular communication [43]. 

## 3. The DYRK Family of Kinases

**Figure 1:**
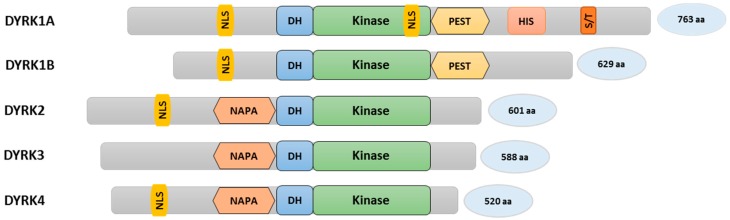
Schematic representation of the DYRK family of proteins: Distinct sequence motifs such as the nuclear localization signal (NLS); DYRK-homology box (DH); a motif rich in proline, glutamic acid, serine, and threonine residues (PEST); a poly-histidine stretch (HIS); a serine/threonine rich region (S/T); a N-terminal auto-phosphorylation accessory region (NAPA); and a conserved kinase domain comprising the structural and functional features of DYRKs.

Dual-specificity tyrosine phosphorylation-regulated kinases belong to the CMGC group of kinases and contain a characteristic sequence motif called the DYRK-homology box (DH box) (Figure 1). YAK1 from budding yeast was the first member of the DYRK family to be discovered [44,45]. There are five members within the mammalian DYRK subfamily and they are categorized into two classes. Class I consists of DYRK1A and DYRK1B (the latter is also known as Minibrain-related kinase (Mirk)), while class II is made up of DYRK2, DYRK3, and DYRK4 [45,46]. The assortment of mammalian DYRKs in the corresponding classes is based on sequence homologies within the conserved kinase domain [46,47]. Certain sequence motifs can only be found in class I DYRKs, such as a C-terminal PEST domain (a region rich in proline (P), glutamic acid (E), serine (S), and threonine (T)) (Figure 1). The PEST sequence is known to act as a signal for rapid protein degradation [48]. However, to our knowledge, this function has not been formally proven in DYRKs. DYRK1A protein stability is regulated through the ubiquitin/proteasome system, but involves an N-terminal region [49]. DYRK1A is the only family member containing a poly-histidine stretch (13 consecutive histidine residues) and a region enriched in serine/threonine residues (S/T-rich region) [50,51]. The poly-histidine stretch promotes the targeting of DYRK1A to nuclear speckles which are enriched with pre-mRNA splicing factors regulating the splicing machinery [51,52]. Other elements, such as nuclear localization signals (NLS), can be found in many DYRKs. On the other hand, only class II DYRK kinases contain a N-terminal auto-phosphorylation accessory region (NAPA) domain, which is thought to be required for tyrosine auto-phosphorylation specifically in class II DYRKs, although DYRK2 lacking the NAPA domain has been shown to auto-phosphorylate itself under in vitro conditions [47,53,54,55]. Further differences include the extent of the respective N- and C-termini (Figure 1). In general, DYRK family members are known to regulate protein stability, cell proliferation, and differentiation. These events are mediated by the phosphorylation of DYRK recognition sites in target proteins. The consensus sequence motif consists of Ser or Thr followed by Pro in position +1. Furthermore, an arginine residue at position −2 or −3 relative to Ser/Thr seems to be preferred (RxxS/TP or RPxS/TP), although a considerable degree of divergence to this consensus has also been noted [47,56].

The activation loop of DYRK kinases contains a conserved YXY sequence, the phosphorylation of which leads to the activation of full enzymatic activity. Members of the DYRK family auto-phosphorylate the second tyrosine residue in order to be fully activated and then phosphorylate substrates in trans on Ser/Thr residues, hence they are known as Dual-specificity tyrosine-regulated kinases [45,47,56]. The auto-activation mode of DYRKs is in contrast to MAP kinases, where an upstream kinase is needed for the phosphorylation of the activation loop [47]. The tyrosine specificity of DYRK kinases is thought to be lost once the protein is fully translated and only the Ser/Thr specificity on target proteins remains [53,57].

## 4. Class I DYRKs: DYRK1A and DYRK1B

### 4.1. The DYRK1A Kinase

DYRK1A is a nuclear kinase, but can also be found in the cytosol. It represents the most studied member of the DYRK family, which is due to its presumed involvement in the Down syndrome (DS, OMIM #190685). DS is one of the most common genetic defects in humans with an estimated incidence of about 1 in 1000 live births worldwide and is caused by the complete or partial duplication of human chromosome 21 (trisomy 21) [58,59]. In humans, the *DYRK1A* gene is located on chromosome 21 (21q22.13), which is part of the so-called *Down-Syndrome Critical Region* (DSCR) [60]. Genes present within the DSCR (21q22.1–22.3 encompassing 33 genes) are thought to account for the development of DS, characterized by a general intellectual impairment, characteristic craniofacial dysmorphologies, and congenital heart disease [60,61,62]. 

Despite the fact that upstream modulators of DYRK1A kinase activity exist [63,64], the prime determinant of DYRK1A protein function is considered to be its overall protein amount making it very sensitive to gene dosage. An altered copy number of the *DYRK1A* gene in mammals or of its orthologous gene, *minibrain (mnb)* in *Drosophila*, impedes with the proper development of the central nervous system [65]. Different studies with the trisomic DS mouse model Ts65Dn or cells derived from Down syndrome patients [66] have shown that an increased kinase expression affects neurogenesis and neuroblast proliferation, and results in impaired behavioral phenotypes. Genetic overexpression of *Dyrk1A* in mice leads to behavioral and cognitive impairment and neuronal alterations [67,68,69]. In contrast, loss of function of *Dyrk1A* or *mnb* results in significant brain size reduction in mice [70], flies [65,71], and men [72].

Intriguingly, a recent study identified *DYRK1A* loss-of-function mutations which are associated with impaired dendritic and spine growth, cortical development, and the pathophysiology of autism [73]. The exact mechanisms underlying DYRK1A’s effects on dendritogenesis and neurogenesis remain open, but might involve its role in actin regulation [36,74,75,76,77]. Furthermore, DYRK1A has functions in synaptogenesis and synaptic vesicle endocytosis [74,78]. Haplo-insufficiency of *DYRK1A* is associated with the development of autosomal dominant mental retardation-7 (MRD7) (OMIM #614104), a syndrome characterized by primary microcephaly, facial dysmorphism, and behavioral problems [79]. Also, *DYRK1A* expression might be epigenetically misregulated in the William-Beuren region duplication syndrome (WBS) (OMIM #609757). WBS phenotypes commonly include craniofacial anomalies and cognitive deficits ranging from mental retardation to autism [80,81]. 

The documented evidence of DYRK1A functioning in brain development suggests that it interacts with embryonic signaling pathways such as Hedgehog, which is known to be crucial for neuronal specification in the neural tube, hippocampal neural stem cell maintenance, and the development of the cerebellar cortex [82,83]. Mice with a genetic *Shh* knockout present with Cyclopia [84], and inactivating mutations in the human *SHH* gene cause holoprosencephaly (OMIM #236100), a common form of structural malformation of the developing brain hemispheres [85,86,87]. In contrast, human patients suffering from the Hh-activating Gorlin syndrome (Basal Cell Nevus Syndrome, OMIM #109400) have an increased brain size [88]. 

### 4.2. DYRK1A as a Regulator of (Neuronal) Hedgehog Signaling

**Figure 2:**
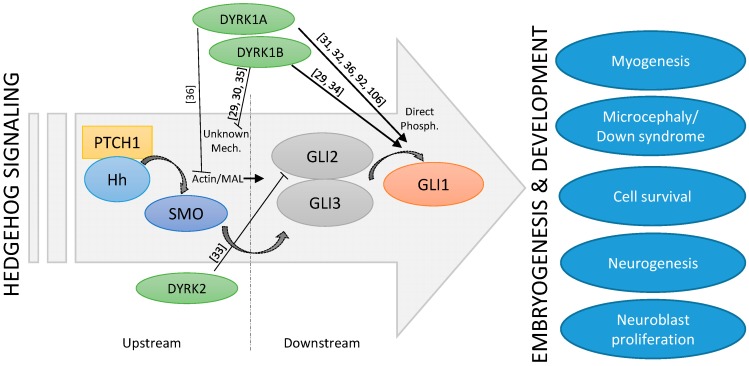
Schematic depiction of the cross-talk between Hh signaling and DYRK kinases.

The fact that both Hh signaling and the DYRK1A kinase have such important roles in embryonic brain development suggests that they might be functionally linked. The exact interplay between DYRK1A and Hh signaling seems to be complex and stimulatory, and inhibitory functions have also been described (Figure 2). Indeed, suppression of Hh pathway activity was seen in cerebellar cells derived from a Down syndrome mouse model [89]. Furthermore, some morphological as well as functional deficits could be ameliorated by the application of a synthetic SMO agonist or by genetic Hh pathway activation [90,91], suggesting a too low level of Hh signaling in DS. Subsequent mechanistic studies revealed that increased levels of the DSCR-localized DYRK1A kinase can dampen Hh signaling [92], most likely through its effect on the actin cytoskeleton and on actin-regulated transcriptional regulators [36]. For instance, DYRK1A can phosphorylate the F-actin stabilizing ABLIM proteins and thereby functionally exert a negative impact on the actin cytoskeleton and on actin-modulated transcriptional co-factors such as MAL (MKL1, MRTF), which also modulate the Hh pathway [36]. This mechanism might explain why DS cerebellar cells display a limited response to Purkinje cell-derived SHH (Figure 2). However, this finding was unexpected as previous reports had proposed a direct activating function of DYRK1A on GLI1. Specifically, DYRK1A can phosphorylate amino acid residues in the N-terminus critical for the nuclear import of GLI1 [31,32,36,92] (Figure 2). As a result, the impact of DYRK1A on Hh signaling might be context-dependent and might also be dictated by the exact mode of pathway activation (ligand/receptor-triggered versus direct GLI1 activation). Further investigations are certainly needed to clarify this point. 

Hypothetically, a physiological connection between Hh signaling and DYRK1A might also exist in the case of neural stem cell (NSC) division. In general, stem or progenitor cells can undergo symmetric or asymmetric types of cell division in order to generate progeny [93]. Hh signaling preferentially supports symmetric cell divisions [94,95]. DYRK1A has also been implicated in signaling aspects during asymmetric versus symmetric neural stem cell division, although the details await further investigation [96,97]. In general, Hh pathway activity has been associated with brain size (see above), which might be caused by its positive effects on neural stem cell pools. Intriguingly, truncation of DYRK1A results in the stimulation of kinase activity [98] and *DYRK1A* gene truncations have been found in human microcephaly [72]. Finally, it is interesting to note that Down syndrome patients have a reduced risk of developing solid cancer. The fact that DYRK1A is capable of suppressing canonical Hh signaling might contribute to its described potential as a tumor suppressor [99,100,101,102,103], in addition to other Hh-independent proposed mechanisms [104,105]. 

In addition to the neuronal effects of DYRK1A, one report exists describing the Hh-related impact of DYRK1A and its physical interactors HAN11 and mDia1 in cultured sebocytes [106]. Overexpression of either HAN11 or mDia1 suppressed GLI1 nuclear localization and activity in reporter assays and slowed the growth of these cells. In the murine embryo, *Han11* is expressed in the developing limb bud (E10.5), together with *Gli1* and *Ptch1*. It is possible that the actin-regulating formin mDia1 is functionally linked to the aforementioned DYRK1A-ABLIM-Actin-MAL-GLI axis, but experimental proof is lacking at this point. 

Three DYRK kinases (DYRK1A, DYRK1B, DYRK2) have been described as regulators of upstream (above GLI transcription factors) and downstream (at the level of GLI transcription factors) Hh signaling. As Hh pathway modulators, they might control important embryogenic and developmental processes, such as myogenesis, neurogenesis, or the pathophysiology of Down syndrome. Literature references are given in square brackets.

### 4.3. The DYRK1B Kinase

The closest relative of mammalian DYRK1A is the DYRK1B kinase, also referred to as MIRK (Minibrain-related kinase). The human *DYRK1B* gene is located on chromosome 19q13.2, a region often amplified in ovarian and pancreatic cancer [107,108]. This kinase has three splice variants (629aa (p69), 601aa (p66), and 589aa (p65)) and is expressed in abundance in human skeletal muscle and testes [109]. Human DYRK1A and DYRK1B proteins are 84% identical in the N-terminal and catalytic domains but show no extended similarity in the C-terminal domain. Human and mouse DYRK1B proteins share 97% sequence similarity [46,110,111,112,113]. In many different cell types, DYRK1B can be found both in the nucleus and in the cytoplasm [114].

### 4.4. DYRK1B in Developmental and Physiological Processes

The observation that DYRK1B is strongly expressed in skeletal muscle argues for a physiological role in muscle function and/or development. Indeed, DYRK1B levels have been shown to be comparatively low in myoblasts, but to increase significantly upon the induction of differentiation [115]. DYRK1B favors myoblast fusion and the subsequent expression of differentiation markers [116]. Furthermore, DYRK1B supports the survival of muscle progenitor (C2C12) cells in culture and of cells from muscle-related tumors such as rhabdomyosarcoma [117,118]. The pro-differentiating effects of DYRK1B on myoblasts are opposite to the effects which Hh signaling exerts on muscle stem cells (satellite cells) and on C2C12 progenitor cells [119]. Here, Hh promotes cell division and blocks differentiation along the myogenic lineage, thereby maintaining the progenitor cell pool. Although it is not clear whether the influence of Hh or DYRK1B occurs exactly at the same developmental stage, currently available data would suggest a primarily antagonistic relationship between these two pathways. Recent work has identified a complex regulatory relationship between DYRK1B and Hh. While DYRK1B dampens Hh signaling initiated by SMO, it also promotes the stability of the GLI1 transcription factor on the other side [29,30,34]. The latter might be mediated by DYRK1B-induced stimulation of the pro-survival PI3K-AKT signaling pathway, a known positive regulator of GLI stability [23,120] (Figure 2). In addition, at least in cultured fibroblasts, Hh pathway stimulation increases DYRK1B protein levels by currently unknown post-transcriptional mechanisms [29], suggesting a feedback loop.

Another example of physiological cross-talk between DYRK1B and Hh might be the differentiation of mesenchymal progenitor cells into adipocytes. Hh signaling has a generally inhibiting impact on adipocytic differentiation, usually redirecting cellular fate towards the osteogenic lineage [121,122,123]. In contrast, DYRK1B favors the in vitro differentiation into adipocytes [35]. This holds particularly true for DYRK1B carrying mutations which were identified in families suffering from an autosomal-dominant form of metabolic syndrome [35], a disease with prominent adipocyte involvement. The mutations found result in misfolding of the DYRK1B protein and in intracellular aggregation [124]. It remains to be clarified how these mutations affect the functional integration of DYRK1B into other signaling pathways, but it is intriguing to note that mutant DYRK1B expression reduced GLI2 levels in cultured adipocytes [35]. It is therefore reasonable to speculate that the suppression of Hh pathway activity contributes to these effects. 

## 5. The Class II DYRKs

### 5.1. The DYRK2, DYRK3, and DYRK4 Kinases

Compared to the DYRK class I members, the class II DYRKs (DYRK2, DYRK3, DYRK4) contain a larger N-terminal region and a shorter C-terminal domain. DYRK2 and DYRK4, but not DYRK3, possess an NLS sequence and all three contain an NAPA (N-terminal autophosphorylation accessory region) domain which is absent in class I DYRKs [47]. The NAPA domain provides a chaperone-like function and transiently converts class II DYRKs into intramolecular tyrosine kinases [54]. Despite lacking an apparent NLS, DYRK3 (also named REDK) is localized in the nucleus in hematopoetic cells [125], whereas DYRK2 is mostly cytosolic, but under conditions of genotoxic stress, it accumulates in the nucleus regulating p53 [126]. DYRK4, which is currently the least studied DYRK family member, displays splice variant-dependent subcellular localization [47]. 

In contrast to DYRK1B, which has been described as an oncogenic kinase in numerous cancer types, DYRK2 can also exert opposite functions and can display tumor suppressive traits. This is brought about by DYRK2’s ability to activate p53-dependent apoptosis following DNA damage [126,127] and by negatively controlling the protein stability of well-established oncogenes such as c-MYC or c-JUN [128]. Phosphorylation-dependent regulation of proteasomal degradation seems to be a recurrent mechanism employed by many if not all DYRK kinases [129,130,131,132,133,134].

### 5.2. Class II DYRKs in Development

In zebrafish, *DYRK2* has been shown to be expressed in lateral somites (mesodermal blocks around the anterior-posterior axis of the developing embryo) and adaxial cells (muscle precursor cells that are adjacent to the notochord and part of the presomitic mesoderm) at an early stage of embryogenesis [135]. Co-localization of *Dyrk2* mRNA and *myogenic differentiation factor D (MyoD)* mRNA was seen in muscle progenitor cells in the posterior compartment of somites. Here, DYRK2 might positively regulate fast twitch muscle differentiation in the early stages of embryonic development [135]. Although the link has not yet been experimentally verified, it is intriguing to note that in contrast to DYRK2, Hh signaling promotes the formation of slow-twitch fibers in zebrafish [136]. Furthermore, mammalian DYRK2 has been shown to negatively regulate Hh pathway activity by phosphorylating and degrading GLI2 [33] (Figure 2). It is therefore reasonable to speculate that DYRK2, through its negative influence on Hh signaling, might impact on the slow/fast-twitch fiber differentiation during muscle development. 

A similarly antagonistic relationship between DYRK2 and Hh signaling might also play a role in *Drosophila*, which encodes three DYRKs: Minibrain/Dyrk1A, DmDyrk2, and DmDyrk3. Recent reports have shown that *DmDyrk2* is expressed in the developing third antennal segment, an anatomical structure responsible for smell, and in the morphogenetic furrow of the developing eye, where it contributes to the development of the visual system [137]. In addition, Hedgehog is a known regulator of morphogenetic furrow progression and ommatidial cell differentiation in the *Drosophila* eye disc [138,139]. If, in analogy to mammals, DmDYRK2 also regulates Hh signaling, it is intriguing to hypothesize that functional DYRK2-Hh cross-talk is involved in the specification of the *Drosophila* eye. 

In comparison to class I DYRKs and DYRK2, the class II family members DYRK3 and DYRK4 show a very restricted expression profile with the strongest expression in erythroid progenitors and testes, respectively [140,141]. As can be assumed from this expression pattern, DYRK3 is involved in erythropoiesis. While *Dyrk3^−/−^* mice surprisingly present without a hematological phenotype, they develop increased numbers of red blood cells under conditions of anemia, suggesting that DYRK3 functions as a negative regulator of erythropoiesis [140]. Therefore, small-molecule DYRK3 inhibitors might be of interest to ameliorate anemic conditions. Although Hh ligands (mostly DHH and IHH) have also been shown to regulate erythropoiesis [142,143,144], it currently remains unclear whether cross-talk between DYRK3 and Hh signaling contributes to this process.

Significantly more work has been done on the function of DYRK3 on the cellular and molecular level, albeit a clear link to Hh signaling has so far not been established. Specifically, DYRK3 impinges on stress-associated mTOR signaling [145], as well as on endocytosis dynamics [146]. Endocytic sorting of SHH and PTCH1 in clathrin-coated vesicles is also critical for proper Hh signaling in signal producing and receiving cells [147,148,149]. However, whether DYRK3 is indeed involved in these steps awaits further experimentation.

Of all the DYRK kinases discussed so far, the least is known about DYRK4. The expression of this family member is strongly restricted to testicular tissue, with a strikingly selective peak of expression in step VIII spermatids [46,141], suggesting a role in male fertility. Surprisingly, however, analysis of *Dyrk4* null animals revealed no aberrant sperm phenotype or defects in male fertility [141]. DYRK4 was shown to be present in the duck ovary, and was more active or upregulated in the high egg production ovaries, which would suggest a hitherto unrecognized role in the female reproductive system, at least in some species [150]. Desert Hh (DHH) signaling also occurs in testes and, at least in certain species, also in ovaries, but the involvement of DYRK4 in DHH-mediated processes is unclear at the moment [151,152,153].

## 6. Conclusions

DYRK kinases are highly conserved during evolution from yeast to humans. Due to the evolutionary diversification, DYRKs might represent the requirement of more critical and specialized functions in vertebrates or might have contributed to this diversification. Multifaceted roles of DYRK kinases have been discussed in this review and their importance in various developmental processes has been stated. As of now, three of five mammalian DYRK kinases have been functionally linked to Hh signaling (DYRK1A, DYRK1B, DYRK2), arguing for a close regulatory connectivity to the developmentally important Hh system. Hence, although DYRK kinases are not absolutely required for Hh signaling, they function as modulators and it is therefore reasonable to hypothesize that they contribute to many Hh-driven steps during embryonic development. Unfortunately, however, more work is needed to provide a clear picture of the exact and tissue-specific cross-talk between DYRKs and Hh, particularly in in vivo settings. As certain DYRKs have a preferred expression in specific tissues (e.g., DYRK1A in neuronal and DYRK1B in muscle tissue), it is reasonable to speculate that the impact on the tissue-selective Hh pathway activity is specified by the respective DYRK enzyme. In other tissues or cell types, where several DYRK kinases are expressed together at comparable levels, a certain degree of functional redundancy might exist, particularly for the class I DYRKs. These questions are important to address in the future in light of the development of small-molecule inhibitors which might lack the necessary specificity and target several DYRKs simultaneously. Complicating the developmental interpretation is the fact that DYRK kinases also modulate other, non-Hh signaling systems, such as, e.g., the NFAT (nuclear factor of activated T-cells) [154] pathway or HIF (hypoxia-inducible factor) signaling [155]. Future work will reveal whether Hedgehog or any of the other signaling systems is particularly important for the physiological impact of DYRK kinases. 

Most evidence for developmental cross-talk between DYRKs and Hh stems from studies on DYRK1A, neuronal development, and the Down syndrome. In addition, available data encourage speculations on DYRK1B and DYRK2 modulating Hh signaling in muscle development and on the involvement of DYRK1B in adipocyte differentiation. In light of recent reports strengthening the concept of Hh-pathway modulation by DYRKs in pathological conditions such as metabolic syndrome or cancer, it will be interesting to see whether future research unveils more cross-talk between this group of kinases and the Hh system in physiological processes. In this review, we have tried to outline the currently available knowledge on the DYRK family of kinases engaging in developmental biology, physiology, and pathology, focusing on its impact on Hh signaling.
